# Using Longitudinal Social Network Analysis to Evaluate a Community-Wide Parenting Intervention

**DOI:** 10.1007/s11121-020-01184-6

**Published:** 2020-11-19

**Authors:** Lisa M. Kleyn, Miles Hewstone, Catherine L. Ward, Ralf Wölfer

**Affiliations:** 1 Department of Psychology, University of Cape Town, Cape Town, South Africa; 2 Department of Experimental Psychology, University of Oxford, Oxford, UK; 3 Department of Experimental Psychology, University of Newcastle, Callaghan, Australia; 4 Deutsches Zentrum für Integrations- und Migrationsforschung, Berlin, Germany

**Keywords:** Parenting, Intervention, Community mobilization, Social influence, Social network analysis

## Abstract

Harsh parenting attitudes and behaviors negatively impact children’s behavior and development, and are linked to heightened levels of violence in children. Parent training programs are effective preventive interventions, but only reach caregivers who attend them. In this study, programs were implemented alongside a community mobilization process, intended to use caregivers’ social networks to disseminate new parenting skills community wide. We used social network analysis to explore whether this intervention, first, increased positive parenting, second, changed social networks of female caregivers (*selection*), and, third, influenced parenting behavior via connections (*socialization*), while controlling for psychiatric morbidity, parenting stress, alcohol misuse, and child’s age. “Colored” Afrikaans-speaking female caregivers (*N* = 235; mean age 35.92 years) in a rural community in South Africa, with children between 1½ and 18 years old, were included in the study; two waves of data were collected (January–April 2016 and June–October 2017). We detected community-wide increases in positive parenting behavior (involvement, supervision, consistent discipline, and reduced corporal punishment). Attending at least one session of a parenting skills training program (*n* = 51; 21.7%) significantly predicted increases in network centrality (i.e., outdegree and indegree). Caregivers appeared to use similar parenting behavior to other caregivers they were connected to within the network, especially when those others attended a parenting skills training program. Overall, the results suggest that the information in the intervention was spread throughout the community through social interactions with program attendees and the community mobilization process. The results also illustrate the value of social network analysis for ascertaining the processes by which the intervention achieved its impact.

**Supplementary Information:**

The online version contains supplementary material available at 10.1007/s11121-020-01184-6.

Violence prevention is recognized as a significant public health concern for low- and middle-income countries (WHO 2016). One effective intervention to reduce violence is parenting skills training programs (WHO 2016; Leijten et al. 2019; Ward et al. 2015) via two mechanisms: reducing violence that children experience from caregivers and preventing development of violent behavior (Coore Desai et al. 2017; Knerr et al. 2013). Parenting skills training programs are, however, expensive to run and they cannot realistically be expected to reach an entire community. Harnessing naturally occurring social influence processes may extend the reach of traditional intervention programs. Here, we present a novel parenting intervention implemented in a deprived community in South Africa, designed to engage an entire community and adjust social norms; its overall aim is to produce widespread and lasting shifts towards positive parenting, and away from harsh, inconsistent parenting. Innovatively, we use social network analysis to explore how the benefits from the intervention components diffuse through the community (Gest et al. 2011).

## The Intervention

This study took place in Touwsranten, a rural, low-income community in the Western Cape Province of South Africa, with an estimated population of 2822 inhabitants and a median household income less than $1384.20 per annum (Statistics South Africa 2015). The community is comprised of two language groups, a larger “Colored” (i.e., mixed-race) Afrikaans-speaking community (*N* = 2218, 78.6%) and a smaller isiXhosa-speaking group (*N* = 604, 21.4%). In spatial terms, the two groups live in distinct sections of the community and constitute two separate social networks (*available online*; see Section 1). Touwsranten provides a suitable setting in which to test this combined approach to improving parenting across a whole community for two reasons. First, it has a low level of population change, which facilitates the assessment of community-level changes over time: since the population does not change much, any changes in parenting in the community are less likely to occur because of new residents bringing in new parenting skills, or former residents leaving, thus reducing the selection bias threat to internal validity. Second, it has clearly defined geographic boundaries and is relatively isolated; this allows for a relatively “pure” manipulation of the independent variable in the form of an intervention, which is unlikely to be “contaminated” by what is being done or occurs in other nearby areas.

The intervention consisted of two components aimed at shifting harsh parenting practices to more positive parenting practices across the entire community: (1) a “community mobilization” process (i.e., a community development process that used social activation methods to mobilize the community around parenting; Parker and Becker-Benton 2016) and (2) four age-specific Parenting for Lifelong Health programs (Ward et al. 2014) rolled out after the mobilization was initiated. In the following sections, we first elucidate these two parts of the intervention, and outline our theory of change. Next, we outline the value of social network analysis for prevention science, and specifically show how it can be used to understand intervention effects.

### Community Mobilization

The community mobilization approach is an effective tool for addressing public health issues in marginalized communities (including Africa: see, for instance, Parker and Becker-Benton 2016; Peltzer et al. 2012) by facilitating shared learning about prevention and reduction of health and social risk factors such as child maltreatment. In Touwsranten, the mobilization process consisted of creating a community-based and community-developed “brand” for positive parenting, alongside a number of child-oriented community activities to support related prosocial values.

### Composition of Parenting Programs

Four evidence-based Parenting for Lifelong Health parenting skills training programs were offered to caregivers to teach them evidence-based, non-violent, effective strategies for parenting (Ward et al. 2019). The parenting programs were the following: (1) Thula Sana, a home visiting program starting during pregnancy that improves attachment between mother and child (Cooper et al. 2009); (2) a cognitive development book sharing program for toddlers (Vally et al. 2015); (3) the Sinovuyo Caring Families Program for children aged 2–9, which increases positive parenting and reduces harsh parenting and child behavior problems (Ward et al. 2019); (4) the Sinovuyo Caring Families Program for parents and teens, which reduces violent discipline, and teen aggression (Cluver et al. 2018).

### Theory of Change

The mechanism of change for the intervention was conceived in terms of diffusion through a social network: caregivers who attended parenting skills training programs are expected to tell their friends and neighbors what they had learned, and so spread the positive parenting concepts beyond the immediate influence of the programs themselves. The community mobilization process would facilitate this diffusion by helping non-attendees become more receptive to the new concepts and by reinforcing concepts once they had been acquired. In this model, the effect of an intervention is linked to the extent to which caregivers can be drawn in as actors to support change processes.

Norms are shared attitudes and behaviors expressing beliefs about the appropriate conduct of individuals; they are strong guides for a range of social behaviors including parenting (Ebersole et al. 2014). Groups establish social norms through the actions of central figures, whose behavior is observed and adopted (Hogg and Reid 2006). Thus, influence in Touwsranten would initially come from the intervention directly, then latterly via indirect influence, whereby the effects of the intervention would be mediated via other central network members (Paluck et al. 2016), and those whom respondents consult on parenting.

### The Value Social Network Analysis Offers Prevention Science

Social network analysis (SNA) describes the links between members of a network in terms of their relationships (Wölfer et al. 2015). In this instance, it can test proposed social influence mechanisms for change (*socialization* and *selection*; Veenstra et al. 2018; Wölfer et al. 2015), that is, shifts in the social network that align with changes in parenting or vice versa. *Selection* processes refer to mechanisms by which people alter their relationships in response to the social context (Veenstra et al. 2018). *Socialization* processes are mechanisms of social influence, and concern how peer relationships can alter individual behaviors (e.g., parenting).

The majority of studies to date which have explicitly used SNA as part of a program evaluation were school-based (e.g., Paluck et al. 2016; Wölfer and Scheithauer 2014), demonstrating that the characteristics of a social network can be exploited to spread social norms, and that these changes are accompanied by changes to the network structure. Several studies have also used SNA to demonstrate the spread of attitudes and behaviors among network members regarding various health-related concerns (Valente 2017).

### Objectives and Hypotheses

The intervention program in Touwsranten was aimed at promoting warmer, more positive parenting in all the individuals within this setting. Due to the community mobilization component, we hypothesized, first, that the intervention would be associated with a mean improvement in parenting practices across all caregivers in the analytic sample, irrespective of program attendance (Hypothesis 1). Second, we theorized that the mechanism for such change would be a significant reorganization of the structure of the network (Wölfer and Scheithauer 2014). As such, we hypothesized that both more positive parenting (Hypothesis 2a) and attending parenting skills training programs (Hypothesis 2b), would be associated with increased network centrality (as measured by multivariate network centrality parameters: indegree, outdegree, closeness, and betweenness). Third, in terms of network structure, we expected that program attendance would be associated both with caregivers nominating more others to speak to about parenting (*selection: covariate ego effects*, Hypothesis 3a) and with caregivers being nominated by more others to speak to about parenting (consistent with a possible increase in their social influence (*selection: covariate alter effects*, Hypothesis 3b). Finally, we conduct further analyses to determine whether connected caregivers behave more similarly (in terms of positive parenting) to each other than would be expected by chance (*socialization: average behavior similarity*, Hypothesis 4). We tested the hypotheses while controlling for risk factors that may undermine parenting (Nkuba et al. 2018).

## Method

### Research Design

The study used a longitudinal design, with two waves of data collection. All measures were assessed at Wave 1 (January–April 2016) and at Wave 2 (July–November 2017). Intervention activities were run continuously between waves of data collection.

### Participants

All residents of Touwsranten who were primary caregivers of children were invited to complete a household survey and questionnaire. In the first wave of data collection, 473 caregivers responded to the questionnaire, of whom the majority (343) were “Colored” Afrikaans-speaking women, who then became the focus of this study, of whom 63 (18.4%) attended at least one parenting skills training program; 108 (22.8%) isiXhosa-speaking caregivers participated, but none attended the parenting programs, which meant we were unable to evaluate the success of the intervention within their network, which was very separate from that of the Afrikaans-speaking network. At Wave 2, 108 (31.5%) caregivers did not participate in the survey. The network boundary for social network analysis was drawn around the 235 caregivers who participated in both waves of data collection (mean age 35.92 years) with children between 1½ and under 18 years old, of whom 51 (21.7%) were attendees (see *participant flow diagram available online*, Section 2)*.*

#### Attrition Analyses and Attendance Selection Bias

Logistic regression analyses indicated that only not attending a parenting skills training program, and no other variable, was associated with dropping out of the study at Wave 2 (*χ*2 (11) = 47.74, *p* < .001; *available online*; see Section 3). The finding that attendees were under-represented in the dropout group was confirmed by chi-squared tests of independence (*χ*2 (1) = 7.20, *p* < .05; 17.5% of attendees and 34.9% of non-attendees). Additionally, there were no systematic differences between attendees and non-attendees in the analytic sample at baseline on the outcomes and covariates used for hypothesis testing (*χ*2 (11) = 12.12, *p* = .277; corresponding group differences *available online*, see Section 4; see also Table 3).

### Procedure

Participants were recruited in a door-to-door survey. In each wave, one caregiver per family was invited to provide informed consent, and then to take part in an interview conducted in a private setting. In Wave 2, the same caregiver was purposely contacted. Data were collected using an Android app (*Mobenzi*). The intervention components’ procedures are discussed below.

#### Community Mobilization

The process unfolded, firstly, by conducting a workshop (in February 2016; followed by a meeting in May 2016) with a small group of caregivers from the community that explored perceptions of positive and negative parenting, reviewed community challenges and potentials for change, developed songs supporting change in the community, and contributed towards the development of a logo to support positive parenting and community change processes. Secondly, we encouraged community engagement through the establishment of a steering committee which held regular meetings over the intervention period to arrange community involvement activities aimed at making the community a safer and more enjoyable space for children (e.g., fund-raisers to rebuild the community park). Many households signed the “Saamstaan” (Standing together) manifesto describing values related to change in Touwsranten, and displayed stickers with the logo on the doors of their homes. The parenting program attendees were invited to participate in the community mobilization aspect.

#### Parenting Programs

The first parenting program (Parenting for Lifelong Health for Young Children) was implemented on the 30th of March 2016, and altogether the four programs were run in total 16 times over the duration of this study. Programs lasting between 8 and 12 weeks were rolled out continually during the intervention period. Caregivers were notified about roll-outs through door-to-door invitations and all were welcome to participate. To enhance program accessibility, community members were trained to deliver the programs, and there was no need for costly materials.

### Measures

#### Social Network Data

The caregivers’ social network was elicited based on a peer nomination procedure. The caregivers were asked to nominate up to five female caregivers (with children under 18 years old) whom they “talk to about parenting *in* the community of Touwsranten”. Caregivers were assigned unique identifiers to disambiguate caregivers with identical names.

#### Parenting Behavior

To account for the fact that parenting behavior differs as a function of children’s age, two age-specific questionnaires were administered in the present study at both waves. Both questionnaires assess positive (i.e., warm, consistent) parenting skills. The Alabama Parenting Questionnaire (APQ, 42 items, for children aged 6–18; Shelton et al. 1996), specifically designed to assess parenting associated with child aggression and confirmed as a valid and reliable measure of parenting in diverse contexts (Elgar et al. 2007), comprises five subscales: (a) Positive Parenting Behavior (example item: “You play games or do other fun things with your child”), (b) Parental Involvement (e.g., “You help your child with his/her homework”), (c) Poor Monitoring and Supervision (reverse scored, R; e.g., “Your child stays out in the evening past the time he/she is supposed to be home”), (d) Inconsistent Discipline (R; e.g., “The punishment you give your child depends on your mood”), and (e) Corporal Punishment (R; e.g., “You slap your child when he/she has done something wrong”). Items are rated using a 5-point Likert-like scale, 1 (*strongly disagree*) to 5 (*strongly agree*).

The Parenting Young Children Scale (PARYC, 14 items; McEachern et al. 2012) assesses the frequency of parenting behaviors relevant for the caregivers of children younger than six, reliably measures parenting behavior in South Africa (Lachman et al. 2017), and comprises two subscales: (a) Supporting Positive Behavior (e.g., “How many times in the past month did you teach your child new skills?”) and (b) Setting Limits (e.g., “How many times in the last month did you stick to your rules and not change your mind?”). Items are rated on a 7-point rating scale, 1 (*never*) to 7 (*almost daily in the past month*).

The main outcome measure is a standardized parenting score constructed by using Wave 1 scores for the whole community on the parenting questionnaires as reference values (Parenting Summary Statistic). Each parent is assigned a *z*-score based on their APQ or PARYC score, with the Wave 1 mean score of the whole community as the reference sample (Fryar et al. 2012), to establish how the analytic caregiving community relates to the community as a whole (*details available online*; see Section 5); higher values indicate improved positive parenting behavior. This statistic allows comparison of parenting across the age-appropriate and validated scales (APQ: older children, PARYC: younger children), thus increasing the sample size and the statistical power of the analytic models.

#### Risk Factors that May Undermine Parenting

The Parenting Stress Index-Short Form (PSI-SF, 36 items; Abidin 1990) screens for stress in the parent-child relationship (e.g., “my child is not able to do as much as I expected”), yields high levels of validity and test-retest reliability in South Africa (Potterton et al. 2007), and has three subscales: Parental Distress, Parent-Child Dysfunctional Interaction, and Difficult Child. Items are rated on a 5-point rating scale, 1 (*strongly disagree*) to 5 (*strongly agree*).

The General Health Questionnaire (GHQ, 28 items; Goldberg and Hillier 1979) is a screening tool for psychiatric morbidity (e.g., “Been feeling nervous and strung-up all the time?”), is a reliable measure of psychiatric morbidity in South Africa (De Kock et al. 2014), and comprises four subscales: Somatic Symptoms, Anxiety, Social Dysfunction, and Depression. Items are rated using a 4-point rating scale, 1 (*better than usual*) to 4 (*much worse than usual*).

The Alcohol, Smoking and Substance Involvement Screening Test (ASSIST, 7 items; (Humeniuk et al. 2010) provides a measure of respondents’ alcohol intake (e.g., “Have you ever tried to control, cut down or stop using alcoholic beverages?”) and has proved a reliable measure in diverse contexts (Mertens et al. 2014). Questions are answered on a frequency scale which ranges from *never* (= 0) to *daily or almost daily* (non-zero values vary by item). The total score (0–36) can be interpreted in terms of alcohol use risk category: low (0–10), moderate (11–26), and high (27+).

#### Community Mobilization Awareness

Caregivers were asked to indicate if they participated in the community mobilization by selecting which from a list of 16 statements (e.g., “You have attended a meeting held by the *social activation group*,” *“*You talk about the *social activation group* meetings at home,” “You have a positive parenting T-shirt”) were applicable to them*.* The caregiver’s score was the total number of statements selected.

### Statistical Analysis

Data were analyzed in four stages using IBM SPSS Statistics version 24, and R version 3.4.3 (Rcore Team 2013). Preliminary analyses assessed whether the data met the primary assumptions necessary for parametric data analysis procedures. Caregivers who answered the relevant questionnaire sections are included in analyses, but numbers necessarily differ because not all caregivers answered all questionnaire sections at both waves.

#### Analysis of Variance

First, we tested Hypothesis 1, whether parenting behavior became more positive, using a 2 (attendance: attending vs not attending) × 2 (time: Wave 1 vs Wave 2) mixed-model analysis of variance (ANOVA), with the second factor within-subjects; partial-eta was squared (${\eta}_p^2\Big)$ to measure effect size. Second, we tested Hypotheses 2a and 2b about network structure using multivariate analysis of covariance (MANCOVA). To explore modifications in the caregiver network, we investigated group differences regarding change parameters (Table 1). We used a 2 (attendance) × 2 (baseline parenting: above vs below average parenting score at Wave 1) × 2 (parenting change: increase vs decrease in positive parenting) between-subjects MANCOVA, controlling for baseline centrality parameters.

**Table 1:** Key centrality parameters

Centrality parameters	
Indegree	The number of incoming ties: The caregivers who are nominated most frequently by their peers have the highest indegree centrality.
Outdegree	The number of outgoing ties: The caregivers who nominate the most others have the highest outdegree centrality.
Betweenness	A measure of the extent to which a caregiver connects otherwise-separate groups of caregivers.
Closeness	The mean number of “hops” from a caregiver to each other caregiver in the network.

#### Social Network Analysis

Network structure is conceptualized in terms of four centrality measures that each differ conceptually, offering slightly different insights into network dynamics. Changes in both these centrality parameters and network positions can influence behavior dynamics and vice versa. The key centrality measures that will be utilized are summarized in Table 1.

Third, we characterized the network structure at Waves 1 and 2 in terms of density, reciprocity, and transitivity. We calculated density both as a percentage of the theoretical maximum: *E*/(*n* × *e*_max_) where *e*_max_ is the number of possible nominations and in terms of standard density figures: *E*/*n*(*n* – 1). We explored reciprocity and transitivity using the relative frequencies of the possible dyads and triads within the network.

Fourth, we separated selection and socialization effects using advanced statistical techniques such as longitudinal stochastic actor-based modelling approaches, including Simulation Investigation for Empirical Network Analysis (SIENA; Ripley et al. 2011). SIENA is a computer program for the analysis of SNA data that is specifically designed for exploring the co-evolution of network and behavior dynamics. SIENA parameters are expressed as logged odds of the evaluation parameters, in other words, the odds that a connection is formed or maintained (or a behavior increased); they denote the magnitude of change over time related to individual and group network characteristics.

To assess *selection*, we examined whether program attendees nominated more others (*covariate ego effect*; Hypothesis 3a), and were nominated by more others (*covariate alter effect*; Hypothesis 3b), while controlling for psychiatric morbidity, stress, alcohol use, and their child’s age. To assess *socialization*, we first examined whether caregivers adopted the parenting behavior of the caregivers they nominated (*average similarity effect*; Hypothesis 4). Next, we assessed the influence of attendee connections on parenting behavior (*alter’s covariate total effect on behavior*) to further explore the relationship between centrality (in terms of attendance status) and influence on parenting behavior, while controlling for general changes in parenting behavior. The direct effect of program attendance on parenting behavior and standard control variables (*available online*; see Section 6) was also included in the model.

## Results

### Descriptive Statistics

Of the caregivers in the analytic sample, 98.7% (*n* = 232) reported being aware of and receiving the community mobilization component: 21.9% were attendees (*M*_awareness_ = 8.68, *SD* = 4.78) and 78.1% were non-attendees (*M*_awareness_ = 6.56, *SD* = 4.06). The survey results indicated that more than half of community members (61.7%; 71.2% of attendees; 59.0% of non-attendees) had heard of the steering committee and around a fifth (25.1%; 42.3% of attendees; 21.4% of non-attendees) had participated in group activities and signed the manifesto. Descriptive data for the network of caregivers are presented in Table 2.

**Table 2:** Key variables of interest at Waves 1 and 2 (analytic sample)

					Wave 1			Wave 2		
						95% bootstrapped CI^b^			95% bootstrapped CI	
Variable name			*n*^a^	Possible range	*M*	Low	High	*M*	Low	High
Demographics	Parent age		235	-	35.92	34.31	37.41	37.54	36.06	39.12
	Child age		235	< 18 years	9.25	8.66	9.84	10.27	9.66	10.84
Risk factors	Parent stress		235	– 6 to 180	83.05	80.51	85.74	83.46	81.17	85.86
	Psychiatric morbidity		235	– 8 to 112	39.98	38.85	41.90	41.83	40.42	43.30
	Alcohol use		122	0–32	7.82	8.01	10.85	8.48	6.81	8.96
Parenting^c^	Standardized parenting behavior total^c^		227	-	0.04	− 0.09	0.16	0.32	0.21	0.42
APQ	Total score		170	1–5	3.66	3.58	3.75	3.74	3.67	3.81
	Inconsistent discipline		170	1–5	2.83	2.73	2.95	2.63	2.52	2.73
	Involvement		170	1–5	3.99	3.88	4.11	3.84	3.73	3.95
	Positive parenting		170	1–5	4.50	4.40	4.58	4.37	4.26	4.47
	Poor monitoring and supervision		170	1–5	1.83	1.73	1.93	1.76	1.67	1.85
	Corporal punishment	Spank	170	1–5	2.92	2.70	3.14	2.40	2.19	2.59
		Slap	170	1–5	1.23	1.14	1.34	1.13	1.05	1.23
		Hit with belt	170	1–5	1.62	1.44	1.81	1.30	1.19	1.42
PARYC	Total score		42	1–7	4.80	4.50	5.10	5.09	4.81	5.37
	Setting limits		42	1–7	4.59	4.25	4.94	4.92	4.51	5.32
	Supporting positive behavior		42	1–7	5.01	4.69	5.32	5.27	4.92	5.59

^a^Of caregivers in the analytic sample, 227 answered questions about a child over 1½ at baseline, of those 212 caregivers answered questions about focus children that were consistent (i.e., remained in the same age category) from Wave 1 to Wave 2: 42 were aged 1½–5 years (19.8%) and 170 were aged 6–18 years (80.2%)

^b^There is no guarantee that the bootstrapped confidence intervals will coincide with those produced by *t* tests

^c^The parenting summary statistic, a measure used to merge caregivers’ self-reports on their behavior with children (younger and older)—calculated with reference to the baseline parenting levels throughout the whole community at Wave 1

*Z*-scores were calculated separately for APQ and PARYC by comparison with the appropriate reference distribution. The scores in this table represent the parenting behavior reported by the caregivers in the study who remained consistent across two waves

### Effects of the Intervention on Parenting Behavior

**Fig. 1:**
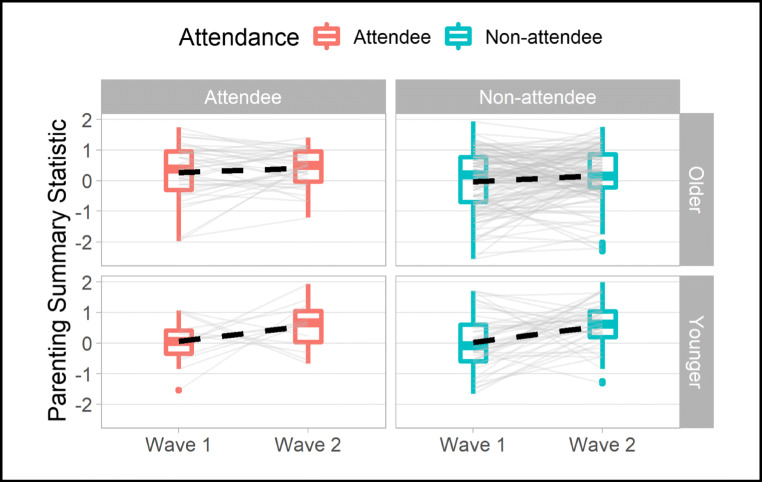
A graphical depiction of improvement in parenting from Wave 1 to Wave 2. Note: Change in Parenting Summary Statistic broken down by child age and caregiver attendance status. Faint gray lines show individual caregivers’ trajectories from Wave 1 to Wave 2, while boxplots characterize the distribution of scores and the thick dashed black line shows the mean trajectory for each group

We started our analysis procedure by assessing whether the intervention brought about a community-wide shift towards warm, positive parenting. Parenting behavior across the community became more positive from Wave 1 to Wave 2 with scores for both the APQ and PARYC increasing from Wave 1 to Wave 2: *Mw1*_*APQ*_ = 3.66 [95% bias-corrected, accelerated confidence interval 3.57, 3.74], *Mw2*_*APQ*_ = 3.74 [3.66, 3.81]; *Mw1*_*PARYC*_ = 4.80 [4.50, 5.10], *Mw2*_*PARYC*_ = 5.09 [4.81, 5.37]. As shown in Table 2, the subscales most substantially influenced by the intervention were supporting positive behavior and reduced corporal punishment. To test the overall effect statistically, we used the standardized positive parenting score so that we could include parents of both younger and older children in the analysis: *F*(1, 225) = 16.21; *p* < .001; ${\eta}_p^2$ = .067; *Mw1* = 0.04 [− 0.10, 0.15], *Mw2* = 0.33 [0.20, 0.46], and confirmed Hypothesis 1 (with an effect size of *d* = 0.32). There was, however, no evidence that attending a program resulted in greater increases in positive parenting scores, *F*(1, 225) = 0.101; *p* = .750; ${\eta}_p^2$ < .001, *M*_attendee improvement_ = 0.30 [− 0.00, 0.60], *M*_non-attendee improvement_ = 0.31 [0.15, 0.47], nor was there evidence that attendees had systematically different parenting scores from non-attendees, *F*(1, 225) = 3.36; *p* = .068; ${\eta}_p^2$ = .015 (see Fig. 1). Further analyses indicated that improvements were primarily driven by caregivers with children in the younger age group (*F*(1, 40) = 4.72; *p* = .036; ${\eta}_p^2$ = .11, *d* = 0.49; full analysis *available online*; see Section 7). Furthermore, a positive association was found when regressing the parenting change scores on community mobilization dose received (*β* = 0.05, *p* = .003,${R}_{adj}^2$ = .03).

### Effects of the Intervention on the Caregiver Network

#### Network Characterization

We then looked to see how the network changed during the course of the intervention. The Wave 1 network consisted of 676 nominations, resulting in a mean degree (i.e., the mean number of incoming and outgoing ties for each caregiver in the network) of 2.88 (*SD* = 1.72) per caregiver. At Wave 2, 746 nominations were made with a mean degree of 3.17 (*SD* = 1.92), of which 164 nominations remained consistent across waves and 582 new nominations were formed. Thus, there was an increase in network density from Wave 1 (.012; 58% of the theoretical maximum density) to Wave 2 (.013; 64%): caregivers reported communicating about parenting with more network members. The majority of ties were asymmetric at Wave 1 (69%) and Wave 2 (83%), and the main triadic structural mechanism of interest, transitivity, increased from 0.087 to 0.126. A paired samples *t* test indicated a significant increase in outdegree (*Mw1* = 1.50, *SD* = 1.28; *Mw2* = 1.65, *SD* = 1.61: *t*(1, 234) = 2.045, *p = .*042); a nonsignificant increase in indegree (*Mw1* = 1.51, *SD* = 0.82; *Mw2* = 1., *SD* = 0.88: *p = .*234); and a significant 7% increase in the number of attendees nominated (*Mw1* = 1.15, *SD* = 0.82; *Mw2* = 1.59, *SD* = 0.86: *t*(1, 234) = − 2.846, *p = .*005).

#### Network Modifications

**Fig. 2:**
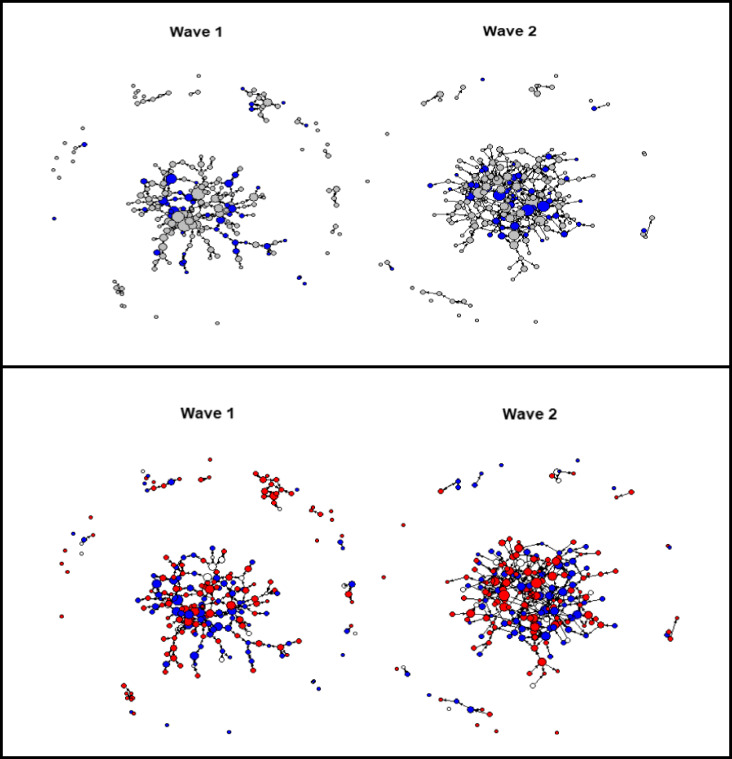
Indegree centrality change by program attendance (blue) and non-attendance (gray) and outdegree centrality change by parenting becoming more positive (red) or more harsh and inconsistent (blue). Note: The change in indegree centrality (size of nodes) of caregivers that attended parenting skills training programs (blue) versus those that did not (gray). The size of the blue nodes increased substantially in the Wave 2 network, indicating that caregivers who attended parenting skills training programs became more important and embedded in the caregiver network. Note: The change in outdegree centrality (the size of the nodes) of caregivers that reported improvement (red) and deterioration (blue) in parenting behavior at Wave 2. The red nodes at Wave 1 indicate where caregivers were situated in the network before the intervention. The size of the red nodes increased substantially in the Wave 2 network, indicating that caregivers who reported improved parenting became more central and influential in the caregiver network. Caregivers demarcated by white nodes did not provide consistent data across waves (due to their children in the younger children at W1 moving into the older category at W2)

We next asked whether caregivers’ behavior could predict the changes to their role in the network. The increase in positive parenting behavior was associated with significant increases in the multivariate network centrality parameters (Pillai’s Trace Parenting Change, *F*(4, 197) = 2.70, *p* = .032). Means for each contingency are presented in Table 3. Thus, there was an association between caregivers who reported improvements in parenting behavior and their engagement with the caregiver network, thereby increasing their potential for disseminating parenting information to others and vice versa (confirming Hypothesis 2a). The univariate test results indicate that improvements in parenting behavior were associated with an increase in outdegree centrality, suggesting that these caregivers may have become more willing to select other caregivers to speak to about parenting. The significant relationship between improvements in parenting behavior and outdegree centrality is depicted in the change in the network structure shown in Fig. 2. As expected, the group main effect revealed that the multivariate network centrality parameters also changed significantly in the attendee group, Pillai’s Trace Attendance, *F*(4, 197) = 3.89, *p* = .005, confirming Hypothesis 2b (see Fig. 2). The univariate test results show a significant association between attendance and both outdegree and indegree centrality indicating that program attendees became more inclined to discuss parenting with others and vice versa.

**Table 3:** Predictors of network change

Attendance status	ΔPositive parenting	ΔIndegree	ΔOutdegree	ΔBetweenness	ΔCloseness
		*μ (σ)*	*μ (σ)*	*μ (σ)*	*μ (σ)*
Attendee (*n* = 47)	Increased (*n* = 24)	1.00 (2.75)	0.50 (1.14)	74.5 (248.2)	1.73 (2.30)
	Decreased (*n* = 23)	0.30 (1.40)	0.26 (1.14)	72.5 (139.4)	1.38 (2.06)
Non-attendee (*n* = 165)	Increased (*n* = 103)	0.08 (1.88)	0.16 (1.01)	85.9 (316.2)	1.46 (2.13)
	Decreased (*n* = 62)	0.00 (1.79)	− 0.15 (1.17)	119.2 (388.5)	1.05 (2.14)
Attendance group *F*(1, 200)		5.29*	5.83***	0.22	0.55
ES (Cohen’s *d*)		0.14	0.31	0.04	0.05
Baseline parenting *F*(1, 200)		0.02	3.70	0.67	2.79
ES (Cohen’s *d*)		0.04	0.12	0.04	0.02
ΔParenting *F*(1, 200)		0.96	5.68***	0.32	0.58
ES (Cohen’s *d*)		0.09	0.21	0.04	0.01
Attendance group x baseline parenting *F*(1, 200)		1.91	1.01	2.18	0.08
Attendance group x ΔParenting *F*(1, 200)		1.13	0.01	0.22	0.01
Baseline parenting x ΔParenting *F*(1, 200)		0.08	3.75	0.02	0.61
Attendance x baseline parenting x ΔParenting *F*(1, 200)		0.71	0.07	0.17	0.20

^a^Coded as “0” non-attendee and “1” attendee

^b^Coded as “0” below average parenting at baseline and “1” above average parenting at baseline; descriptives of the change in network parameters and the corresponding univariate outcomes are reported separately for attendees and non-attendees, based on parenting change (i.e., improvement). There were no caregivers for whom there was no change. *p < .05; ***p < .001

#### Longitudinal Network Effects

We wanted to further explore the impact of caregivers’ behavior on network evolution as well as, whether parenting behavior spread through the network through caregiver-to-caregiver contact using SIENA. First, we assessed whether the network was sufficiently stable and dynamic to submit to the iterative simulation approach used by SIENA. The rate parameter, fixed output of the iterative simulation in SIENA, indicated that significant network changes between the waves enabled caregivers to alter their network and that the model was interpretable (*e*^5.33^ = 206.44; Table 4). The maximum convergence ratio of the model was satisfactory at < .20. The T-ratios for individual parameters also achieved a satisfactory convergence of below .10 (Ripley et al. 2011). Overall, the model fits the data well enough that its parameters could be meaningfully interpreted.

**Table 4:** SIENA estimates of selection and influence effects on parenting behavior in caregiver network (Wave 1 and Wave 2)

	Estimate	*SE*	|*t* value|^a^	Odds ratio
Network dynamics^b^				
Network rate	5.33	0.62	8.65*	206.85
Structural effects				
Outdegree (density)	− 3.14	0.09	− 35.38*	0.04
Reciprocity	2.04	0.23	8.77*	7.69
Balance	2.49	0.31	8.00*	12.06
Covariate ego effects				
Psychiatric morbidity	0.01	0.01	0.96	1.01
Parenting stress	0.00	0.00	− 1.20	1.00
Alcohol misuse	0.00	0.01	− 0.12	1.00
Child age	− 0.02	0.01	− 1.25	0.98
Program attendance	0.06	0.02	3.54*	1.07
Covariate alter effects				
Psychiatric morbidity	0.00	0.01	− 0.57	1.00
Parenting stress	0.00	0.00	− 1.63	1.00
Alcohol misuse	0.00	0.01	− 0.29	1.00
Child age	0.02	0.01	1.65	1.02
Program attendance	0.03	0.01	2.23*	1.03
Covariate similarity effects				
Psychiatric morbidity	− 0.02	0.35	− 0.07	0.98
Parenting stress	− 0.28	0.39	− 0.70	0.76
Alcohol misuse	− 0.33	0.21	− 1.59	0.72
Child age	0.77	0.25	3.03*	2.16
Program attendance	0.07	0.11	0.65	1.07
Behavior dynamics^c^				
Behavioral rate	3.18	0.77	4.13*	24.05
Shape effects				
Linear tendency	0.49	0.25	1.92*	1.63
Quadratic tendency	− 0.30	0.28	− 1.05	0.74
Socialization effect				
Average similarity	1.82	0.91	2.00*	6.17
Parenting: effect from attendance	0.15	0.55	0.28	1.17
Parenting: total attendee connections	0.47	0.18	2.55*	1.60

^a^Reported as absolute values

^b^Parameter estimates for network dynamics are log-odds of (*P*(form new connection) + *P*(maintain existing connection)) / (*P*(sever connection) + *P*(maintain non-connection))

^c^Parameter estimates for behavior dynamics are log-odds of *P*(increased behavior score) / *P*(decreased behavior score)

*p < .05

We also examined the standard controls which describe how the network behaved in the absence of the covariates of interest. There was a negative tendency to form random ties (*e*^−3.14^ = 0.04). However, caregivers tended to create reciprocal connections (*e*^2.04^ = 7.69), and to connect with others that made similar network choices (*e*^2.49^ = 12.06). This indicates that caregivers often met other caregivers to speak to about parenting through their existing network connections.

Second, we assessed the impact of caregivers’ behavior on network choices by interpreting the selection effects present in the model. Attending sessions of the parenting skills training program was associated with an increased likelihood of both forming new network ties or maintaining old ties (covariate ego effect; *e*^0.07^ = 1.07, providing support for Hypothesis 3a) and receiving new network ties (covariate alter effect; *e*^*0*.03^ = 1.03, providing support for Hypothesis 3b), suggesting that attendees may have had more opportunities to influence other caregivers in the network. Interestingly, child age similarity (i.e., being more inclined to speak to each other about caregiving practices if one’s children are a comparable age) was also associated with network evolution. Furthermore, these results remained the same when controlling for reported psychiatric morbidity, parenting stress, and alcohol use (none of which influenced the network structure).

Third, we assessed whether parenting behavior spread through the network via caregiver connections by interpreting the socialization effects present in the model. The SIENA model confirms that the mean positive parenting behavior increased slightly from baseline to Wave 2 (linear tendency; *e*^0.49^ = 1.62), and caregivers appeared to adopt the parenting behavior of their caregiver connections (average similarity effect; *e*^1.82^ = 6.17; Hypothesis 4). The significant socialization effect suggests that diffusion occurred, indicating that caregivers’ parenting scores exerted influence on the parenting scores of others to whom they are connected. Moreover, there was no evidence that attendees differed from non-attendees in their overall pattern of parenting behavior change (*e*^0.15^ = 1.17). However, having more attendee connections appeared to increase the likelihood of adopting positive parenting behavior (*e*^0.47^ = 1.60).

## Discussion

This parenting intervention was implemented in an attempt to address South Africa’s need for cost-effective, early violence prevention strategies (Ward et al. 2015). The results are encouraging, suggesting that the intervention may have brought about community-wide change in behavior and social network organization. First, there was a mean increase over time in positive parenting practices across all caregivers in the analytic sample, irrespective of their program attendance (confirming Hypothesis 1). Second, post-intervention, caregivers whose parenting became more positive became more central to the network (as measured by multivariate network centrality parameters; confirming Hypothesis 2a), and caregivers who attended parenting skills training programs became more central to the network (confirming Hypothesis 2b). Third, program attendance was associated both with caregivers nominating more network members (confirming Hypothesis 3a), and them being nominated by more network members over time (confirming Hypothesis 3b), thus increasing their potential influence in the community. Finally, caregivers’ social network position was associated with their change in parenting behavior (confirming Hypothesis 4). We discuss these results in terms of the main hypotheses, first considering the effects of the intervention on parenting behavior, then in terms of network modifications and longitudinal network effects. Finally, we acknowledge some limitations of this study and identify future directions for research.

### Effects of the Intervention on Parenting Behavior (H1)

The caregivers’ self-reports, 1 year after the intervention, indicated that their parenting improved, on average, across the entire community (confirming H1). Attendees did not show greater improvements in parenting behavior than non-attendees. However, there was a main effect of wave, suggesting that attendees and non-attendees improved at the same rate—plausibly due to diffusion of the intervention’s effects (Paluck et al. 2016). Having more exposure to the widespread community mobilization process was associated with improvement in parenting behavior, providing a further explanation of why the differences between attendees and non-attendees were less pronounced than they would have been in an intervention with a randomized controlled design (e.g., Cluver et al. 2018).

### Network Modifications (H2a and H2b)

Attending parenting skills training program sessions and reporting improvements in parenting behavior were associated with increases in multivariate network centrality (confirming H2a and H2b). This result suggests that attendees increased their network centrality and potential for higher social influence within the network. However, it is also plausible that caregivers that were interested in growing their network were more likely to attend program sessions. Similarly, while we expect that improvements in parenting behavior lead to increases in centrality or influence, we cannot rule out the reverse. Our design does not offer a direct test of the relationship between centrality and influence: we measure conversations about parenting but cannot say definitively who is influenced by whom. However, because we can consider attendees as likely to transfer knowledge from the programs (irrespective of any reciprocal influence) we are able to assume that the directionality of knowledge shared during social interactions is from attendee to non-attendee. Therefore, we equate both higher indegree and outdegree centrality of attendees with greater potential influence (also supported by the significant socialization effect reported below). Plausible explanations for the increase in multivariate network centrality associated with improvements in parenting behavior and program attendance include the following: (a) caregivers who grew in competence may also have gained confidence in their parenting ability and thus become more inclined to reach out to discuss parenting with others; and (b) these more competent caregivers may have become recognized as trusted individuals with valuable information to share on this topic, and who were sought out by other caregivers (e.g., Venkatramanan and Kumar 2011).

### Network Effects (H3a, H3b, H4)

The SIENA analyses revealed a general propensity for caregivers who attended the programs to become more embedded in the parenting network due to an increase in either incoming (confirming H3a) or outgoing (confirming H3b) ties. This may have been partly a result of the process by which the programs operate, that is, group problem-solving of parenting issues. Consequently, caregivers attending programs may be more inclined to reach out to other caregivers. The increasing outdegree centrality of attendees could also account for the significant covariate alter effect (i.e., being nominated by more network members over time) due to the question used to construct the social network (which asked who respondents “talk to about parenting” and thus does not indicate whether a particular person was the originator or the recipient of communication).

For behavioral evolution, the rate function describes the average number of changes in behavior between measurement points. The significant socialization effect (i.e., average similarity effect) indicates that caregivers’ parenting behavior was influenced by the parenting behavior of caregivers they spoke to about parenting (confirming H4). Thus, there is a tendency of caregivers to grow more similar to one another possibly in response to processes such as peer modelling and pressures to conform (Gest et al. 2011). Moreover, the likelihood of caregivers adopting positive parenting behaviors appears to increase as the number of attendee connections increase, possibly due to the dissemination of positive parenting skills taught at the programs.

Collectively, these results indicate that caregivers who attended parenting programs became more connected within the caregiver network than did non-attendees, and caregivers influenced the behavior of those to whom they were connected—particularly, if they attended a parenting skills training program. Therefore, due to the increased likelihood of attendees speaking about parenting, and the significant socialization effect, it appears that on average attendees may be influencing the behavior of other network members more than non-attendees do.

Previous studies have made use of social network analysis to reveal the effects of specific intervention programs to bring about wide-scale improvements in targeted behaviors, in both school settings (e.g., bullying: Wölfer and Scheithauer 2014) and health domains (e.g., smoking: Schaefer et al. 2013). These interventions often target highly connected individuals, and use them as vectors via whom the benefits of the intervention can be delivered to the wider community. In the present study, attendees were self-selecting, and were not significantly more central (and potentially influential) in the caregiver network than non-attendees prior to the intervention. Importantly, attendees increased their potential for social influence after the intervention, suggesting that effective social vectors can be made as well as found—a significant departure from the current literature on dissemination of information.

### Limitations and Future Directions

Notwithstanding the effects reported, we acknowledge that this study has some limitations. First, we conducted the intervention in a single community. Moreover, while we noted that the stable population and geographical isolation of this community made it particularly suitable for testing the intervention, these characteristics could also reduce the external validity of the program. Ideally, future research would take the form of a larger-scale randomized controlled trial, across more sites, and with a no-treatment control group.

Second, as acknowledged, almost a third of caregivers did not participate in the second wave, raising the issues of whether our sample was biased, and findings could even be generalized to the whole community studied. This threat to external validity was, however, mitigated by findings of our attrition analyses. The only study variable on which we found a difference between the analytic sample and respondents who dropped out was that program attendees were over-represented in the analytic sample; but as noted, attendees were few in number, and when we tested for differences between attendees and non-attendees that remained part of the analytic sample, we found no systematic differences. Moreover, those who had not participated in the parenting programs would continue to have been exposed to the community mobilization. Overall, these tests suggest that our results can be generalized to the Touwsranten population in general.

Third, it might be argued that, given the intervention aimed at promoting more positive parenting, caregivers asked at Wave 2 might have felt under subtle influence to report an improvement in parenting. However, the network analyses showed that caregivers who reported improvements in parenting behavior also increased their potential for social influence in the parenting network, suggesting that these may not have been purely socially desirable responses. Future research should also analyze children’s reports of caregiving behavior to address this issue.

Fourth, based on the conventional interpretation, some of the effects reported are rather small in size (i.e., smaller than .20; Gignac and Szodorai 2016). These effects are, however, in line with the usual effect sizes obtained for prevention programs—typically characterized by small to moderate effect sizes (Leijten et al. 2019). Nevertheless, small effects can accumulate into more significant changes over time and can have important influences on behavioral choices individuals make (Funder and Ozer 2019). The changed self-reports of particular parenting behaviors identified in this study are an essential prerequisite for sustained behavioral changes (Valente 2017), particularly if positive parenting norms become more widely embedded in the community.

The present research has demonstrated the promise of this particular intervention strategy for preventing violence against children across whole communities. These results therefore suggest a means (community mobilization) and a process (norm change via social networks) for amplifying the effects of individually oriented parenting skills training programs, which may be more cost effective than simply delivering stand-alone programs. Furthermore, our research adds to a body of literature exploring social networks and program evaluation, by suggesting that the mechanism for community-wide change was associated with changes in social network structure.

## Supplementary Information

ESM 1 (DOCX 6678 kb)
