# Supplementary material for: Using Longitudinal Social Network Analysis to Evaluate a Community-Wide Parenting Intervention
Source: Prev Sci. 2020 Nov 19;22(1):130–43. doi: 10.1007/s11121-020-01184-6 (PMC7762743; doi:10.1007/s11121-020-01184-6)
Supplement: Supplementary file 1 — (DOCX 6678 kb) [file 11121_2020_1184_MOESM1_ESM.docx]

# Supplementary Material

[Supplementary Material 1](#_Toc39472385)

[Section 1. isiXhosa Caregivers 2](#_Toc39472386)

[Section 2: Final Analytic Sample Flow Diagram 3](#_Toc39472387)

[Section 3: Attrition Analyses 4](#_Toc39472388)

[Section 4: Program Attendance Selection Bias 5](#_Toc39472389)

[Section 5: Parenting Measure Construction 6](#_Toc39472390)

[Section 6. SIENA Model Specification 11](#_Toc39472393)

[Section 7. Effects of the Intervention on Parenting Behavior 12](#_Toc39472394)

[References 13](#_Toc39472395)

## Section 1. isiXhosa Caregivers

The community is comprised of two language groups, a larger ‘Colored’ (i.e., mixed-race) Afrikaans-speaking community (*N* = 2218, 78.6%), and a smaller isiXhosa-speaking community (*N* = 604, 21.4%). In spatial terms, the two groups live in distinct areas (see Figure S1.1) and constitute two separate social networks (see Figure S1.2).

*Figure S1.1*. Aerial View of the Community of Touwsranten

| 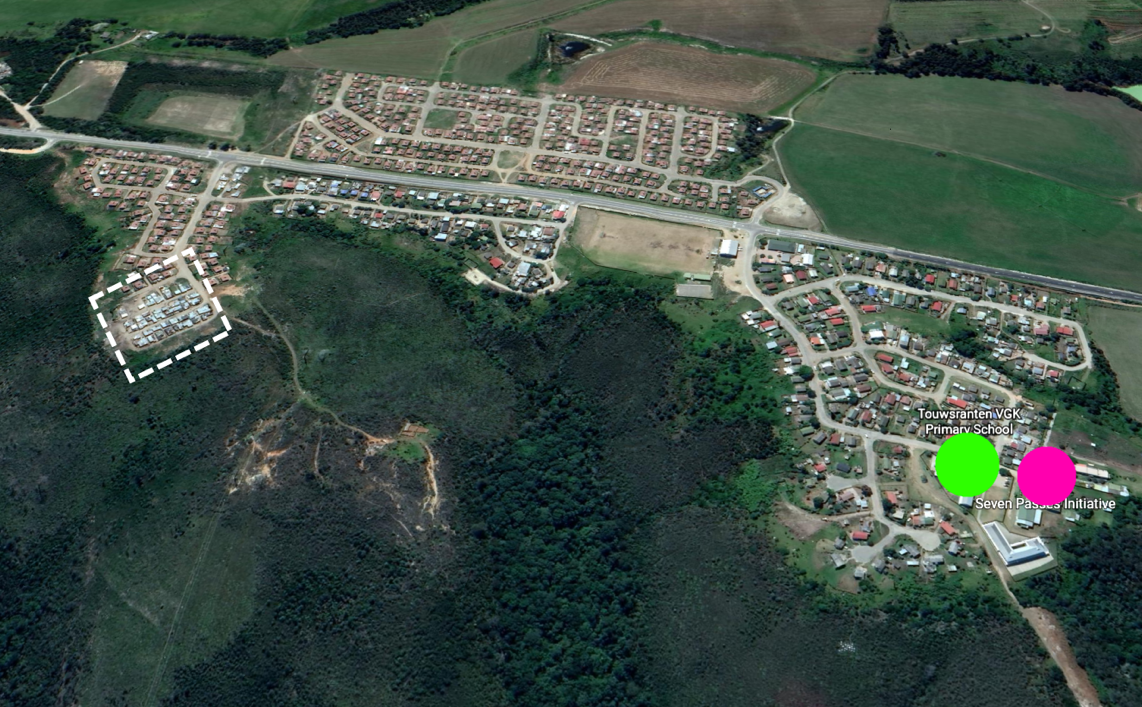  *Note*. The white dashed area demarcates where the isiXhosa-speaking community members reside. The other homes have Afrikaans-speaking inhabitants. The Seven Passes Initiative is marked with the pink circle, while the Touwsranten Primary School is marked with the green circle. |
| --- |

*Figure S1.2.* Afrikaans (red) and isiXhosa-speaking (blue) caregiver networks

| 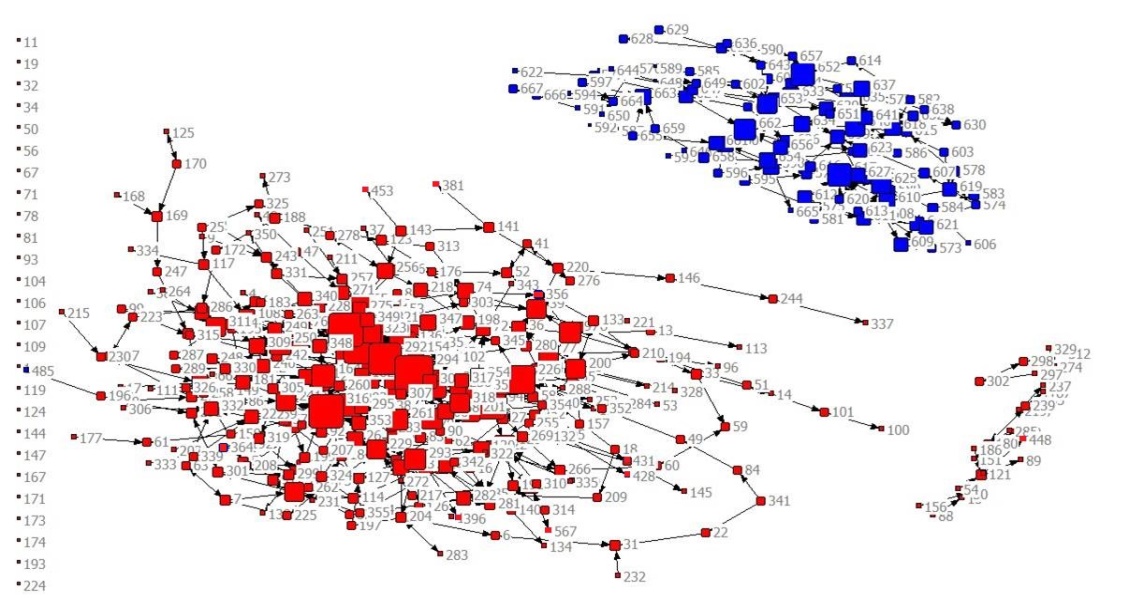 |
| --- |
| *Note*. Node sizes indicate indegree centrality at baseline. |

## Section 2: Final Analytic Sample Flow Diagram

*Figure S2.1.* Final analytic sample (denoted by black oblong) and exclusions.


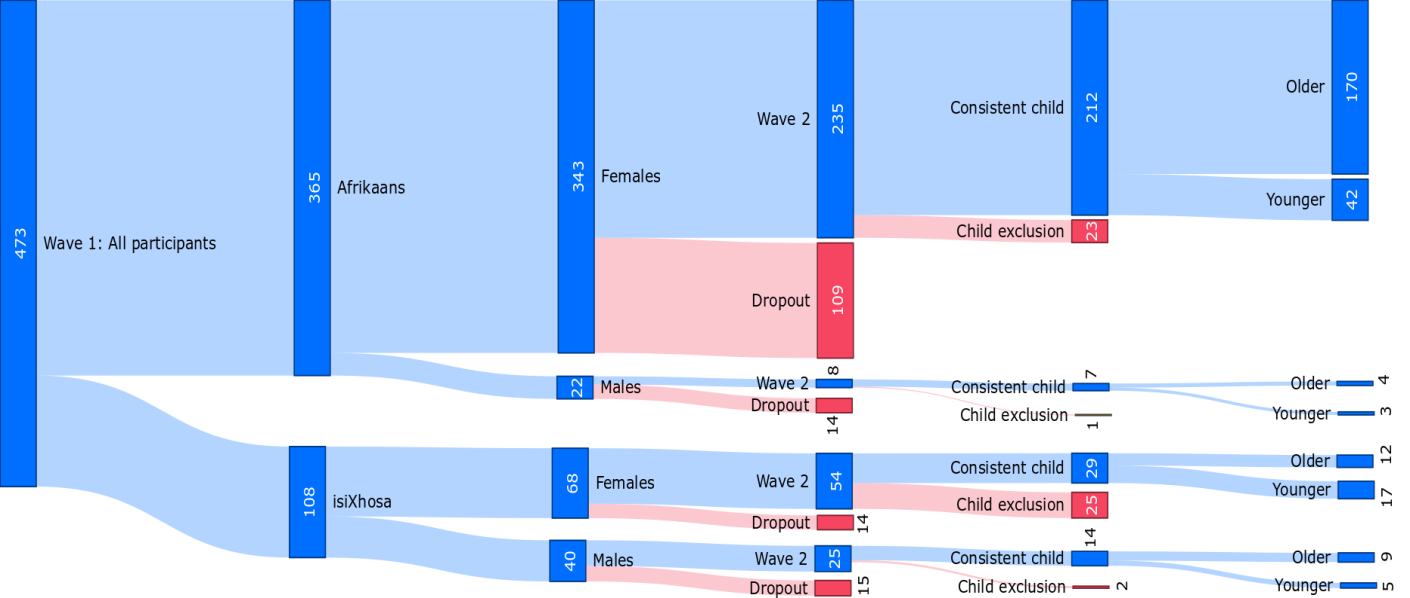


##

*Note:* The network boundary for social network analysis was drawn around these 235 caregivers: 227 of these caregivers in the analytic sample answered questions about a child over 1½ at baseline, and 212 caregivers answered questions about focus children that were consistent (i.e., remained in the same age category) from Wave 1 to Wave 2. Of the consistent focus children 42 were aged 1½-5 years (19.8%) and 170 were aged 6-18 years (80.2%). Reasons for child exclusion included: children aging into a new age group, or caregivers answering questions about a different focus child.

## Section 3: Attrition Analyses

A logistic regression analysis indicated that no variables used for hypothesis testing, other than not attending a parenting skills training program, were associated with dropping out of the study at Wave 2 (χ2 (11) = 47.74, *p* < .001; see Table S3.1). The result indicating that attendees were under-represented in the dropout group was confirmed by a chi-squared test of independence (χ2 (1) = 7.20, *p* < .05; 17.5% of attendees and 34.9% of non-attendees). It should be noted that this was attrition from the study and not from the intervention: although those who refused participation in the study were more likely to be those who had not participated in the parenting programs, they would continue to have been exposed to the community mobilization.

Table S3.1

Logistic Regression Models on dropping out of the study between waves 1 and 2 against the variables used for hypothesis testing.

| Coefficients: | Dropout | Remain | Estimate | SE | Wald | p |
| --- | --- | --- | --- | --- | --- | --- |
|  | *µ* (*σ*) | *µ* (*σ*) |  |  |  |  |
| (Intercept) |  |  | -1.61 | 1.18 | -1.37 | .170 |
| Parent Age | 34.31 (10.16) | 35.92 (12.05) | -.01 | .003 | -2.11 | .063 |
| Child Age | 9.25(4.92) | 9.21 (4.63) | -0.02 | 0.03 | -0.58 | .561 |
| Parent Stress | 84.06 (20.23) | 83.05 (18.95) | 0.00 | 0.01 | 0.03 | .974 |
| Alcohol Severity | 7.93 (9.67) | 7.82 (8.86) | 0.04 | 0.28 | 0.13 | .894 |
| Psychiatric Morbidity | 40.21 (11.45) | 39.98 (10.98) | -0.01 | 0.01 | -0.72 | .474 |
| Parenting Summary Statistic | -0.08 (1.10) | 0.04 (0.95) | -0.08 | 0.13 | -0.61 | .540 |
| Program Attendance^a^ | .00 (.00) | .21 (.41) | -0.40 | .072 | -0.02 | .997 |
| Network Centrality Parameters | | | | | | |
| Indegree | 1.51 (1.20) | 1.50 (1.28) | 0.01 | 0.09 | 0.16 | .871 |
| Outdegree | 1.50 (1.31) | 1.51 (0.82) | -0.12 | 0.13 | -0.98 | .328 |
| Betweenness | 485.21 (1181.67) | 452.80 (1005.63) | 0.00 | 0.00 | 0.38 | .701 |
| Closeness | 10.42 (1.81) | 10.17 (1.82) | 0.16 | 0.09 | 1.88 | .060 |
| χ2 | 47.74, df = 11, p < .001 | | | | | |
| Nagelkerke Pseudo$R^{2}$ | 0.22 | | | | | |

*Note.* Dropout refers to caregivers that dropped out of the analytic sample at Wave 2; while Remain refers to caregivers that form part of the analytic sample. The dependent variable in this analysis is coded as “0” remain and “1” dropout. ^a^Coded as “0” non-attendee and “1” attendee.

## Section 4: Program Attendance Selection Bias

All the caregivers who completed a survey at Wave 1 were made aware of, and invited to participate in, the parenting programs; some caregivers chose to attend, and others not to. This raises the possibility of selection bias (in the sense of systematic differences between these two naturally occurring groups), and therefore a logistic regression analysis was conducted to determine whether systematic differences existed between attendees and non-attendees. At baseline, there were no group differences on the outcomes and covariates used in further hypothesis testing: parenting behavior, parental stress, general health, alcohol use, child age, and the four centrality parameters (χ2 (11) = 12.12, *p* = .277; see Table S4.1 for the corresponding group differences).

Table S4.1

Logistic Regression Models on attendance at parenting skills training programs against the variables used for hypothesis testing.

| Coefficients: | Attendees | Non-attendee | Estimate | SE | Wald | p |
| --- | --- | --- | --- | --- | --- | --- |
|  | *µ (σ)* | *µ (σ)* |  |  |  |  |
| (Intercept) |  |  | -3.12 | 1.67 | -1.87 | .062 |
| Parent Age | 34.31 (10.16) | 35.92 (12.05) | -.006 | .003 | -2.11 | .063 |
| Child Age | 8.91 (3.93) | 9.99 (4.62) | -0.07 | 0.04 | -1.71 | .087 |
| Child Gender *^a^* | 1.50 (.51) | 1.54 (.50) | -0.01 | 0.34 | -0.04 | .966 |
| Parent Stress | 83.92 (1.82) | 82.18 (19.23) | 0.01 | 0.01 | 0.84 | .400 |
| Alcohol Severity | 8.12 (9.83) | 7.57 (9.42) | 0.09 | 0.13 | 0.68 | .494 |
| Psychiatric Morbidity | 40.54 (11.96) | 39.41 (11.55) | 0.01 | 0.02 | 0.91 | .362 |
| Parenting Summary Statistic | 0.12 (0.14) | -0.02 (0.71) | 0.40 | 0.21 | 1.93 | .069 |
| Network Centrality Parameters | | | | | | |
| Indegree | 1.50 (1.20) | 1.51 (1.22) | -0.05 | 0.13 | -0.37 | .713 |
| Outdegree | 1.49 (1.15) | 1.54 (1.40) | -0.20 | 0.19 | -1.04 | .298 |
| Betweenness | 519.32 (1136.53) | 386.28 (1004.2) | 0.00 | 0.00 | 1.29 | .199 |
| Closeness | 10.32 (1.82) | 10.02 (1.84) | 0.13 | 0.11 | 1.17 | .242 |
| χ2 | 12.12, *df* = 11, *p* = .277 | | | | | |
| Nagelkerk Pseudo$R^{2}$ | 0.12 | | | | | |

*Note.* The dependent variable has been coded as “0” non-attendee and “1” attendee.

^b^ Coded as “1” male and “2” female.

## Section 5: Parenting Measure Construction

### Reason for creating a parenting summary statistic.

The overarching research question of interest is whether caregivers in an entire community can improve their parenting practices, thereby moving from a harsh parenting style to a more positive approach to parenting. The measures of parenting (i.e., APQ and PARYC) used to evaluate the main intervention program were selected specifically to assess the change in the parenting of children in different age groups (APQ measure: for caregivers with children older than six; PARYC measure: for caregivers with children younger than six), but unfortunately used different response scales (3-point and 7-point, respectively). To avoid loss of statistical power when using sub-samples we assessed change in parenting of very young and older children on the same metric by using a reference population approach: standardized parenting scores at Wave 1 were used to benchmark scores at Wave 2. The reference population approach to interpretation of individuals’ scores is a technique frequently used in anthropometric and economic studies (Vidmar, Carlin, Hesketh, & Cole, 2004; Wang & Chen, 2012). Below we delineate how we used this approach to create a continuous and combined parenting change variable, termed the *parenting summary statistic*.

### Construction of primary outcome measure of positive parenting scores.

The outcome measure is change in the *parenting summary statistic* (*P*), expressed in terms of the standard deviation of the reference sample (all caregivers at Wave 1). This is calculated as follows:

$$\Delta zP_{i} = {zP'}_{i,2}-{zP}_{i,1}$$

where ${zP}_{i,1}$is the standardized (z-score) for the summary statistic for caregiver $i$ at Wave 1, and ${zP'}_{i,2}$ is the comparative score for the summary statistic for caregiver $i$, at Wave 2. The calculation of each of these components is described below. The Wave 1 standardized measure (z-score) is obtained in the usual manner (Field, 2009; Fryar, Gu, & Ogden, 2012):

$$zP_{i,1} = \frac{P_{i,1}-\bar{P_{1}}}{\sigma_{P_{1}}}$$

where $P_{i,1}$ is caregiver$i$’s $P$ score at Wave 1, $\bar{P_{1}}$ is the mean score in the parenting summary statistic at Wave 1, and $\sigma_{P_{1}}$is the corresponding standard deviation. The comparative score is calculated using the Wave 2 parenting summary statistic. To obtain this comparative score, the z-score for the Wave 2 summary statistic is calculated using the population statistics of the reference sample:

$${zP'}_{i,2} = \frac{P_{i,2}-\bar{P_{1}}}{\sigma_{P_{1}}}$$

The comparative score for Wave 2 thus represents where a caregiver would appear within the Wave 1 sample on the basis of their Wave 2 score, while the standardized score for Wave 1 represents where they actually appeared within the Wave 1 sample. Both are expressed in terms of standard deviations of the Wave 1 sample. In other words, a person’s score at Wave 2 indicates where that score would have placed them in the Wave 1 distribution. As such, the Parenting Summary Statistic is not simply a standardized score at Wave 2. We specifically constructed Wave 1 as a reference sample (Fryar et al., 2012). Thus, based on the measurement construction one would be able to determine whether the Wave 2 population either improved or deteriorated with reference to the Wave 1 population.

The scores for both parenting questionnaires were normally distributed throughout the respective populations (i.e., skewness between -2.00 and +2.00, and kurtosis between -7.00 and +7.00), and the variance was comparable across waves (see Figure S4.1; Field, 2009). The APQ skewness was -.63 (*SE* = .16) at Wave 1 and -.73 (*SE* = .18) at Wave 2; kurtosis values were .13 (*SE* = .31) at Wave 1 and .48 (*SE* = .36) at Wave 2. The PARYC weighted average skewness statistic at Wave 1 was -.022 (*SE* = .26), and at Wave 2 was 1.10 (*SE* = .34); and kurtosis values were -.40 (*SE* = .51) at Wave 1 and -.39 (*SE* = .67) at Wave 2. As such, the measures met the normality assumptions which suggest that using z-score standardization to establish a reference population is appropriate to reflect the underlying population.

Several analyses required categorical rather than continuous variables, and to this end we calculated binary variables for key measures. Caregivers were assigned a Baseline Parenting category, coded as 1 or 0, based on whether their standardized parenting score at Wave 1 was above or below the mean of the reference sample. They were also assigned a Parenting Change category based on whether or not their parenting summary statistic was above 0 (= 1) or not (= 0). A representation of this process is shown in Figures S5.1, S5.2 and S5.3.

*Figure S5.1.* Distribution of parenting questionnaire scores.

| 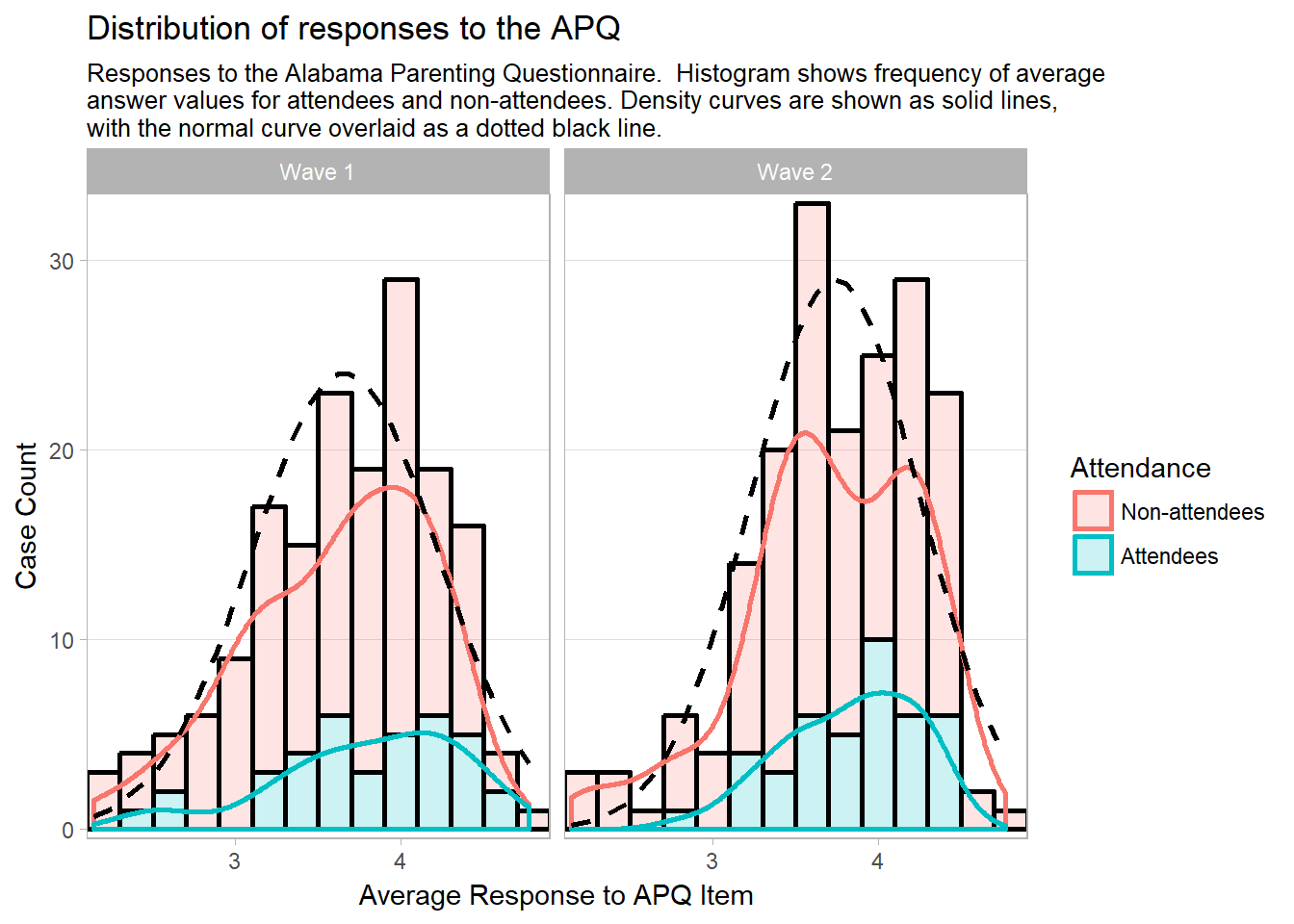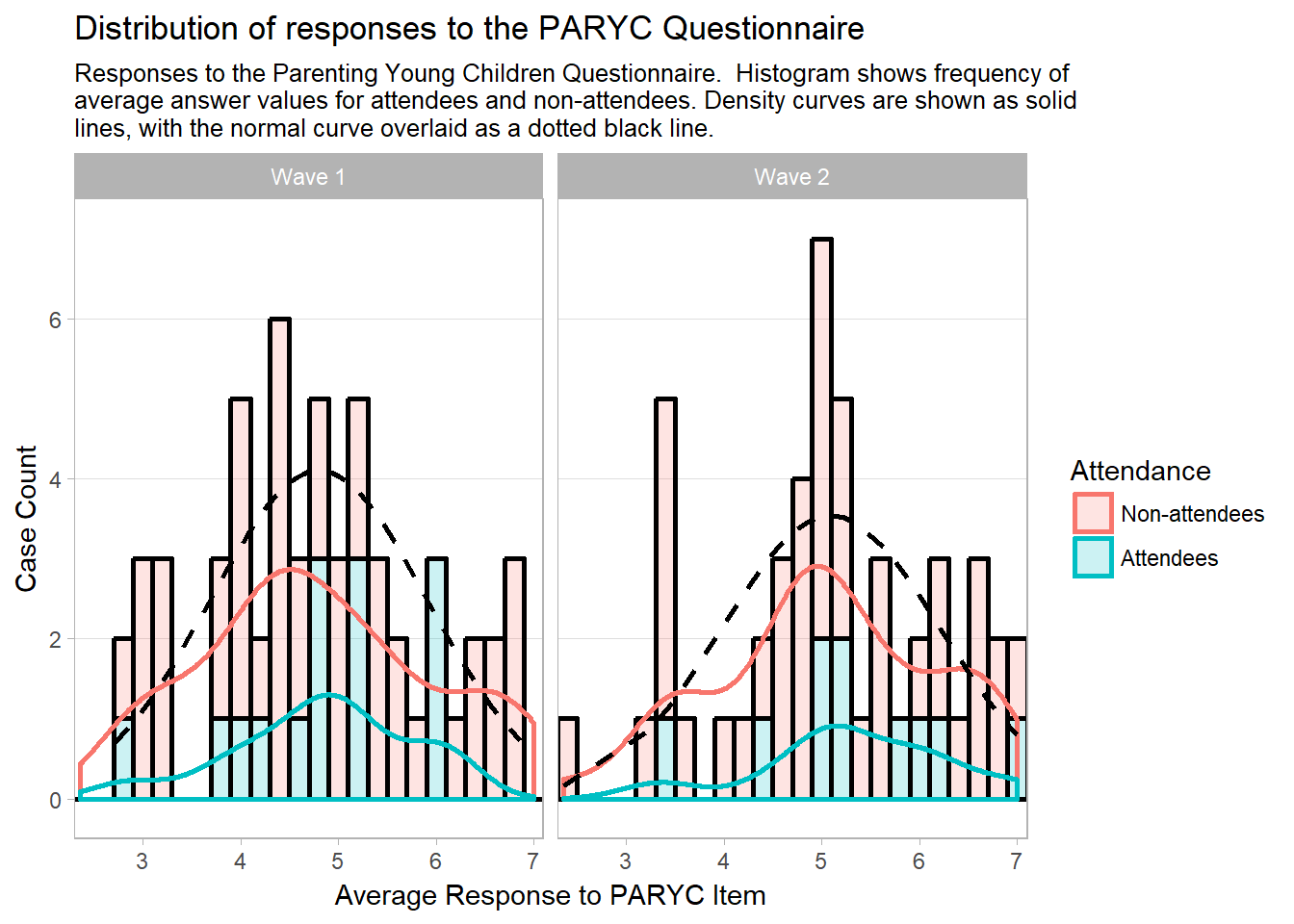 |
| --- |
| *Note*. Histogram shows frequency of average answer values for attendees (blue) and non-attendees (pink) at Waves 1 and 2. Density curves are shown as solid lines, with the normal curve overlaid as a dotted black line. The parenting questionnaire scores are approximately normally distributed, with no major differences between attendees and non-attendees. This indicates that the use of the standardized score is appropriate and unlikely to introduce a systematic bias for or against attendees. Columns show total frequencies for non-attendees and attendees separately. For both questionnaires, higher mean responses indicate more positive parenting. |

*Figure S5.2.* Threshold operation for deriving Baseline Parenting variable.

| 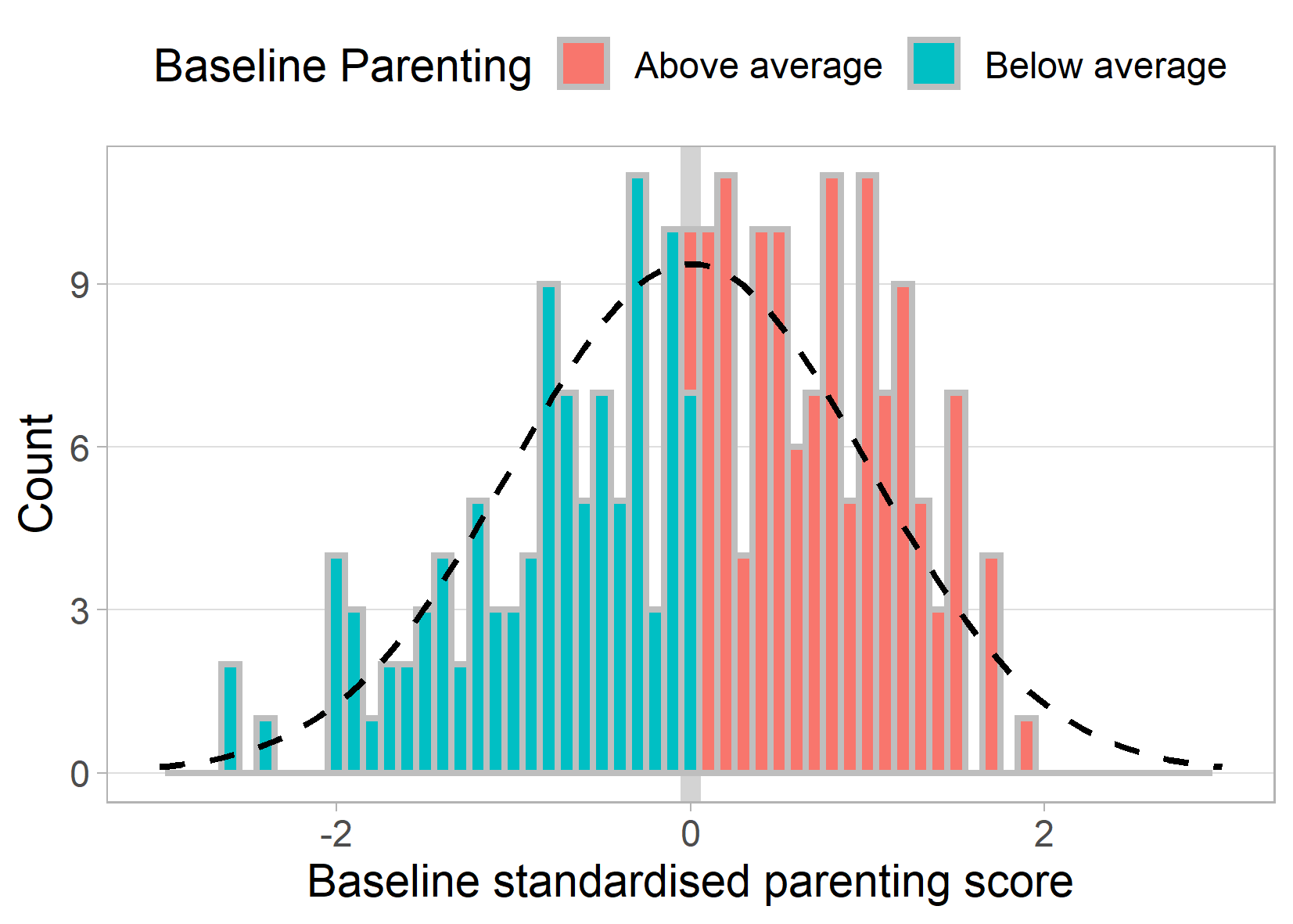 |
| --- |
| *Note*. The Baseline Parenting variable is calculated by assigning a value of 1 to those caregivers with a Baseline Standardized Parenting Score greater than zero, and a value of 0 to others. |

*Figure S5.3*. Threshold operation for deriving the Parenting Change variable.

| 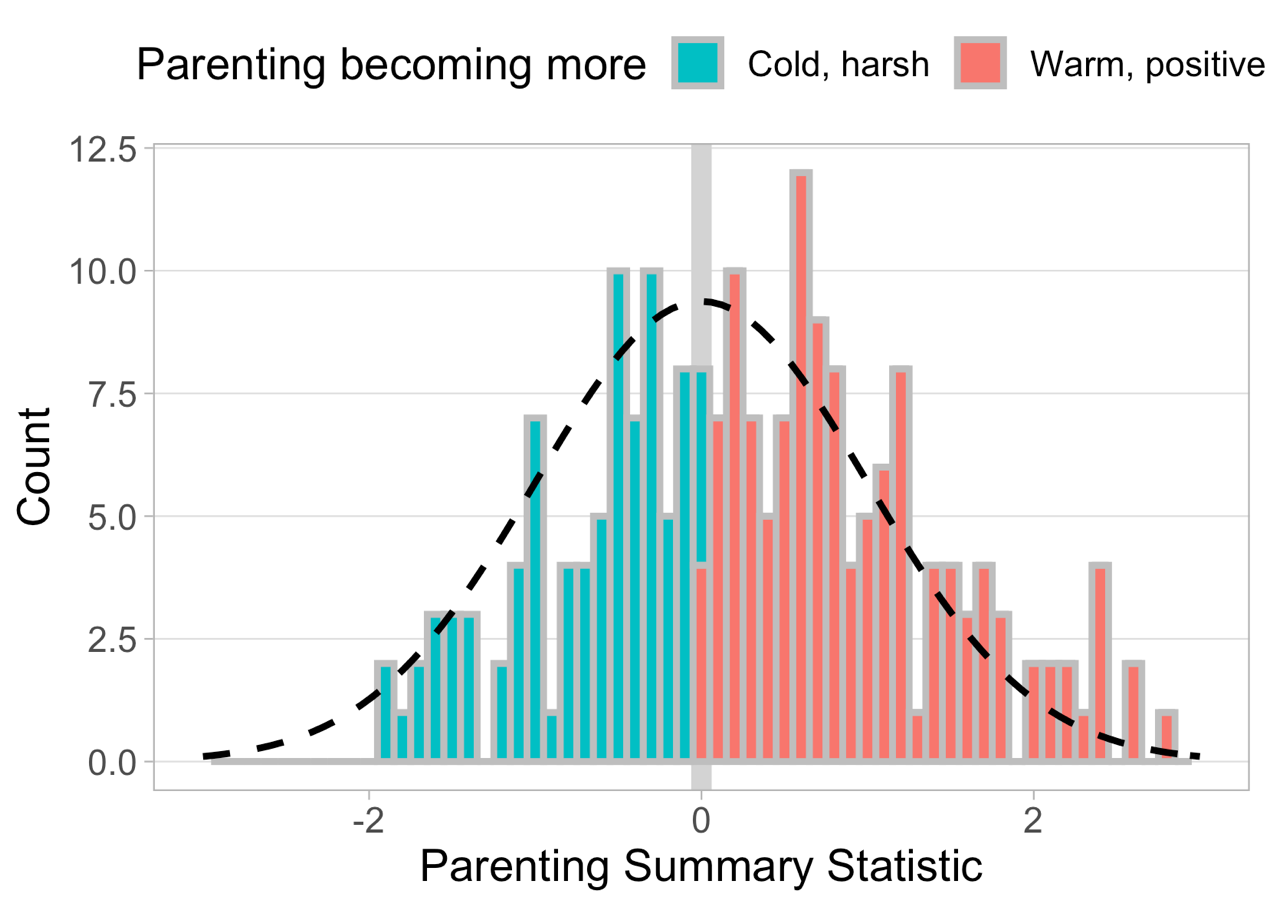 |
| --- |
| *Note*. The Parenting Change variable is calculated by assigning a value of 1 to those caregivers with a Parenting Summary Statistic greater than zero, and a value of 0 to others. |

## Section 6. SIENA Model Specification

When testing selection effects, network dynamics are categorised into two main categories: a) endogenous (i.e., structural network effects), and b) exogenous (i.e., attribute related selection effects). Evidence suggests that the endogenous effects are interdependent, and should be included in the model as control variables to mitigate simpler, more general explanations for changes in selection processes biasing exogenous effects (Ripley, Snijders, Boda, Vörös, & Preciado, 2011). The structural network effects included in the model were: (1) outdegree (density), the tendency for a caregiver to form a tie to a random network member; (2) reciprocal tie formation between caregivers; (3) balance, the tendency to maintain or make new ties with caregivers that make similar network choices (Veenstra, Dijkstra, Steglich, & Van Zalk, 2013).

When testing socialization effects, behavior dynamics are categorised into: a) shape effects (i.e., behavioral tendencies) and b) influence effects. Shape effects are used as important control variables that model the distribution of the behavior under investigation to assess the baseline probability of changes taking place. Influence effects are used to test the likelihood of a specific attribute altering the behavior of others they are directly or indirectly connected to in the network structure (Veenstra et al., 2013). The shape effects included in the model were: a) linear tendency (i.e., an intercept indicating the average tendency towards values on the variables under investigation; Veenstra et al., 2013); and, b) quadratic tendency (i.e., the extent to which extremes of a behavior are self-reinforcing or self-correcting - a positive or negative feedback loop with itself).

## Section 7. Effects of the Intervention on Parenting Behavior

When the data are split by child age, the improvements evidenced in the overall caregiver sample remain consistent for caregivers with younger children, but not for caregivers with older children.

*Caregivers with younger children*. There was a main effect of wave for the weighted total scores (*F*(1,40) = 4.72; *p* = .036; $\eta_{p}^{2}$ = .11), with parenting scores improving from Wave 1 to Wave 2 *Mw1_PARYC_* = 4.80 [95% bias-corrected, accelerated confidence interval 4.50, 5.10], *Mw2_PARYC_* = 5.09 [4.81, 5.37]; *d* = 0.49); there was no effect of program attendance (*F*(1,40) = 0.04; *p = .*848; $\eta_{p}^{2}$ = .001), nor an interaction effect of wave and program attendance (*F*(1,40) = 0.14; *p = .*711; $\eta_{p}^{2}$ = .003). Thus, caregivers with younger children reported improvements at the same rate as non-attendees.

*Caregivers with older children*. For caregivers with older children there was no main effect of wave for the weighted total scores (*F*(1,168) = 2.97; *p = .*087; $\eta_{p}^{2}$ = .000; *Mw1_APQ_* = 3.66 [3.57, 3.74], *Mw2_APQ_* = 3.74 [3.66, 3.81]; *d* = 0.15). There was an effect of program attendance (*F*(1,168) = 4.02; *p* = .047; $\eta_{p}^{2}$ = .023), but no interaction effect between wave and program attendance (*F*(1,168) = 0.025; *p = .*876; $\eta_{p}^{2}$ = .000). The main of effect of program attendance indicates that these caregivers of older children in the attendance group reported greater improvements than non-attendees.

# References

Field, A. (2009). *Discovering statistics using SPSS*. *Sage Publication* (Vol. 58). doi:10.1234/12345678

Fryar, C. D., Gu, Q., & Ogden, C. L. (2012). Anthropometric reference data for children and adults: United States, 2007-2010. *Vital Health Statistics*, *5*(11), 1–40. doi: 10.1186/1471-2431-8-10

Ripley, R., Snijders, T. A. B., Boda, Z., Vörös, A., & Preciado, P. (2011). Manual for RSiena. *University of Oxford; Department of Statistics; Nuffield College.* Retrieved from http://www.stats.ox.ac.uk/~snijders/siena/RSiena_Manual.pdf

Veenstra, R., Dijkstra, J. K., Steglich, C., & Van Zalk, M. H. W. (2013). Network-behavior dynamics. *Journal of Research on Adolescence*, *23*(3), 399–412. doi: 10.1111/jora.12070

Vidmar, S., Carlin, J., Hesketh, K., & Cole, T. (2004). Standardizing anthropometric measures in children and adolescents with new functions for egen. *The Stata Journal*, *4*(1), 50–55. Retrieved from http://ideas.repec.org/c/boc/bocode/s457279.html

Wang, Y., & Chen, H. J. (2012). Use of percentiles and z-scores in Anthropometry. In *Handbook of Anthropometry: Physical Measures of Human Form in Health and Disease* (pp. 1–3107). Springer New York. doi: 10.1007/978-1-4419-1788-1
